# Thrombotic and bleeding events, mortality, and anticoagulant use among 546,656 hospitalized patients with COVID-19 in the United States: a retrospective cohort study

**DOI:** 10.1007/s11239-022-02644-2

**Published:** 2022-04-30

**Authors:** Steve Deitelzweig, Xuemei Luo, Jennifer L. Nguyen, Deepa Malhotra, Birol Emir, Cristina Russ, Xiaoyan Li, Theodore C. Lee, Mauricio Ferri, Danny Wiederkehr, Maya Reimbaeva, Geoffrey D. Barnes, Gregory Piazza

**Affiliations:** 1 Ochsner Clinic Foundation, Department of Hospital Medicine, Ochsner Medical Center, The University of Queensland School of Medicine, Ochsner Clinical School, 1514 Jefferson Hwy, New Orleans, LA 70121 USA; 2 Pfizer Inc., Groton, CT USA; 3 Pfizer Inc., New York, NY USA; 4 Bristol Myers Squibb Company, Lawrenceville, NJ USA; 5 Frankel Cardiovascular Center, University of Michigan, Ann Arbor, MI USA; 6 Brigham and Women’s Hospital, Boston, MA USA

**Keywords:** COVID-19, Thrombosis, Venous thromboembolism, Anticoagulants, Bleeding

## Abstract

This study describes demographics, thrombotic and bleeding events, mortality, and anticoagulant use among hospitalized patients with COVID-19 in the United States. Premier Healthcare Database data were analyzed to identify inpatients with a discharge diagnosis for COVID-19 (ICD-10-CM code: U07.1) from April 1, 2020 to March 31, 2021, and matched historical controls without COVID-19 (inpatients discharged between April 1, 2018 and March 31, 2019). Thrombotic [including venous thromboembolism (VTE)] and bleeding events were based on ICD-10-CM discharge diagnosis codes. Of the 546,656 patients hospitalized with COVID-19, 20.1% were admitted to the ICU, 62.8% were aged ≥ 60 years, 51.5% were male, and 31.0% were non-white. Any thrombotic event was diagnosed in 10.0% of hospitalized and 20.8% of ICU patients with COVID-19 versus (vs) 11.5% and 24.4% for historical controls, respectively. More VTE events were observed in hospitalized and ICU patients with COVID-19 than historical controls (hospitalized: 4.4% vs 2.7%, respectively; ICU: 8.3% vs 5.2%, respectively; both *P* < 0.0001). Bleeding events were diagnosed in 10.2% of hospitalized and 21.8% of ICU patients with COVID-19 vs 16.0% and 33.2% for historical controls, respectively. Mortality among hospitalized (12.4%) and ICU (38.5%) patients with COVID-19 was higher vs historical controls (2.4%, *P* < 0.0001 and 9.4%, *P* < 0.0001, respectively) and higher in hospitalized patients with COVID-19 who had thrombotic events (29.4%) vs those without thrombotic events (10.8%, *P* < 0.0001). VTE and mortality were higher in hospitalized and ICU patients with COVID-19 vs historical controls. The presence of thrombotic events was associated with worse outcomes.

**Supplementary Information:**

The online version contains supplementary material available at 10.1007/s11239-022-02644-2.

## Highlights

- Overall, thrombotic and bleeding event rates among patients with COVID-19 were not higher than matched historical control patients without COVID-19.
- The VTE event rate and mortality were higher among hospitalized and ICU patients with COVID-19 compared with matched historical controls.
- Patients with both COVID-19 and thrombotic events had higher mortality than those with COVID-19 but without thrombotic events.
- Ongoing and future studies investigating optimal anticoagulant regimens that can be used for thromboprophylaxis in patients with COVID-19 are warranted.

## Introduction

Hospitalized patients with coronavirus disease 2019 (COVID-19), especially critically ill patients, may have a higher risk of thrombosis than patients with other acute infections [1, 2]. However, the range of reported incidence of thrombosis among hospitalized patients with COVID-19 is broad, partly due to differences in study design (e.g., definition of thrombosis) and practices (e.g., thromboprophylaxis utilization) within different institutions and regions [3, 4]. For example, studies that evaluated thrombotic events reported their incidence in 7.7–16.0% of hospitalized patients with COVID-19 and 6.7–29.4% of those admitted to the intensive care unit (ICU) [1, 2, 5]. Studies that focused on venous thromboembolism (VTE) have also reported variable rates, ranging from 3 to 23% in non-ICU patients and 8–69% in ICU patients with COVID-19, again due to different systematic screening protocols and thromboprophylaxis patterns [6]. This variability makes it difficult to understand the actual thrombosis risk in hospitalized patients with COVID-19.

Larger studies based on data from many institutions are needed to have a better understanding of the range of thrombotic risk in patients with COVID-19 [3, 7]. Using a nationwide hospital dataset, this study was conducted to describe patient characteristics, thrombotic and bleeding events, mortality, and anticoagulant use among hospitalized patients with COVID-19 in the United States (US).

## Methods

### Data source

Data were obtained through the Premier Healthcare Database (PHD; Charlotte, NC, USA) and PHD Special Release (PHD-SR) developed for COVID-19. PHD is a hospital-based, service-level, all-payer database containing information on inpatient discharges and hospital-based outpatient visits from nonprofit, nongovernmental, community, and teaching hospitals and healthcare systems from rural and urban areas across the US [8].

The PHD-SR provides records starting January 1, 2019, with data becoming available 1–3 weeks from date of discharge, while the PHD provides records starting in 2011, with data becoming available approximately 6 months from date of discharge [8].

Data were extracted from the PHD-SR to identify patients with COVID-19 and from PHD to identify historical controls. The PHD and PHD-SR are compliant with the Health Insurance Portability and Accountability Act and deidentified for patient privacy [8]. Consistent with the use of existing deidentified data, this analysis was exempt from institutional review board approval.

### Patient population

This study included hospitalized patients with a discharge diagnosis for COVID-19, identified using the International Classification of Diseases, Tenth Revision, Clinical Modification (ICD-10-CM) diagnosis code, U07.1, in any position. The sample included patients in the database with the U07.1 code who were admitted to hospitals on April 1, 2020 or later and discharged between April 1, 2020 (when the code was introduced in the US) and March 31, 2021 [9, 10]. Guidelines for using the U07.1 code require a diagnosis of COVID-19 confirmed by a healthcare provider, documentation of a positive COVID-19 test result, or a presumptive positive COVID-19 test result. In the case of COVID-19, confirmation of diagnosis does not require documentation of the type of test performed [9]. Patients with suspected COVID-19 but without the U07.1 code were not included in the study.

A cohort of patients without COVID-19 was included as a historical control. Patients in the historical control group were admitted to the hospital on April 1, 2018, or later and discharged by March 31, 2019. Historical controls were matched to patients with COVID-19 at a 1:1 ratio, separately for all hospitalized patients and those admitted to the ICU. For each patient with COVID-19, one control patient that exactly matched on age, race, ethnicity, sex, US Census Bureau division, admission month, and discharge month was randomly selected for comparison. Due to matching, the number of hospitalized patients with COVID-19 in the matched population was slightly smaller than the number of overall hospitalized patients with COVID-19.

### Demographic and clinical characteristics

Demographic characteristics collected for the study population included age at COVID-19 diagnosis, sex, US Census Bureau division where diagnosis and treatment occurred (e.g., New England), race, and ethnicity.

Comorbid diagnoses were identified based on discharge diagnosis codes in any position. Comorbidities evaluated were atrial fibrillation, cerebrovascular disease, coronary artery disease, hyperlipidemia, obesity, rheumatologic disease, congestive heart failure, diabetes mellitus, hypertension, renal disease, liver disease, chronic obstructive pulmonary disease, peptic ulcer disease, inflammatory bowel disease, peripheral vascular disease, and any malignancy.

Several laboratory values were evaluated, including D-dimer levels. The first, maximum, and minimum test values were evaluated only for hospitalized patients with COVID-19 with available laboratory data. If a patient had only one test, it was used to impute entries for all three values.

### Study outcomes

Thrombotic events were identified based on discharge diagnosis codes in any position and included VTE, disseminated intravascular coagulation, arterial thromboembolism, ischemic stroke, myocardial infarction, and acute coronary syndrome. VTE includes pulmonary embolism and deep vein thrombosis (DVT; including lower-extremity phlebitis, lower-extremity DVT, superior vena cava thrombosis, and upper-extremity DVT). The ICD-10-CM diagnostic codes used to identify thrombotic events can be found in Supplementary Table 1. These codes were selected based on published literature and internal author review [11].

Bleeding events were identified based on discharge diagnosis codes in any position and were categorized into any bleeding, gastrointestinal bleeding, intracranial hemorrhage, and other bleeding. The ICD-10-CM diagnostic codes used to identify bleeding events can be found in Supplementary Table 2. These codes were converted from the International Classification of Diseases, Ninth Revision, Clinical Modification diagnostic codes used in a previous publication [12]. Bleeding was evaluated without differentiation of major and non-major bleeding due to the challenge of determining bleeding severity based on claims data.

Additional outcomes included the length of hospital stay, ICU admission status, shock, and death.

### Anticoagulant use

The use of any anticoagulant, any parenteral anticoagulant (heparin, low-molecular-weight heparin [enoxaparin or dalteparin], and fondaparinux), and any oral anticoagulant (OAC; warfarin, apixaban, dabigatran, edoxaban, and rivaroxaban) was evaluated for hospitalized patients with COVID-19. Patients may have received multiple anticoagulants; each anticoagulant that was prescribed for  ≥ 1 day was considered for the analysis. Anticoagulant use was further assessed by dosage.

### Data analyses

All analyses were conducted using SAS software, version 9.4 (SAS, Cary, NC, USA). For continuous variables, descriptive statistics (i.e., mean, standard deviation, median, and interquartile range [quarter 1, quarter 3]) were summarized. Pooled *t*-tests were conducted for comparisons for equal variances, and the Satterthwaite method was used for *t*-tests of unequal variances. Percentages were calculated for categorical and binary variables, and chi-square tests were used for comparisons. Statistical significance was *P* < 0.05. Due to the large sample size included in this study, differences that were statistically significant but clinically comparable may have been detected.

## Results

### Demographic and clinical characteristics

A total of 546,656 patients hospitalized with a diagnosis of COVID-19 at 875 hospitals across all US Census Bureau divisions were identified in the database. COVID-19 was listed as the primary discharge diagnosis for 62.2% of patients and as the secondary discharge diagnosis for 37.8% of patients. Demographic and clinical characteristics of all hospitalized patients with COVID-19 are listed in Table 1; 62.8% were aged ≥ 60 years, 31.0% were non-white, and 20.1% were admitted to the ICU.

**Table 1:** Demographic and clinical characteristics of hospitalized patients with COVID-19

Characteristics	All COVID-19 Inpatients			All COVID-19 ICU Inpatients			All COVID-19 ICU vs All COVID-19 Non-ICU *P* Value
	Total N = 546,656	Thrombotic event n = 56,015	No thrombotic event n = 490,641	Total n = 110,111	Thrombotic event n = 23,461	No thrombotic event n = 86,650	
Age, years, mean (SD)	63.1 (18.0)	68.3 (14.5)	62.5 (18.3)	64.1 (16.1)	66.6 (13.8)	63.4 (16.6)	< 0.0001
Age group, %							< 0.0001
< 18 years	0.9	0.1	1.0	1.1	0.2	1.3	
18–39 years	11.1	4.2	11.9	7.2	4.0	8.1	
40–59 years	25.1	20.8	25.6	24.7	23.1	25.1	
60–79 years	43.1	50.2	42.2	51.0	55.2	49.5	
≥ 80 years	19.8	24.7	19.2	16.3	17.5	16.0	
Sex, %							< 0.0001
Male	51.5	59.0	50.7	58.7	62.8	57.5	
Female	48.4	41.0	49.3	41.3	37.1	42.4	
Unknown	0.1	0	0.1	0.1	0	0.1	
Race, %							< 0.0001
White	64.3	63.5	64.4	64.1	62.6	64.5	
Black	17.4	18.8	17.2	16.9	17.9	16.7	
Asian	2.5	2.3	2.5	2.4	2.5	2.4	
Other	11.1	11.1	11.1	11.7	12.1	11.5	
Unknown	4.8	4.4	4.8	4.9	5.0	4.9	
Ethnicity, %							< 0.0001
Hispanic	66.7	14.5	17.8	18.3	16.7	18.7	
Not Hispanic	17.5	68.6	66.5	65.5	65.9	65.4	
Unknown	15.9	17.0	15.7	16.2	17.4	15.9	
US Census division, %							< 0.0001
East North Central	15.4	16.9	15.2	16.1	17.4	15.7	
East South Central	7.3	6.4	7.4	6.8	5.4	7.2	
Middle Atlantic	15.9	17.5	15.7	11.5	13.7	10.9	
Mountain	8.0	7.9	8.0	8.1	8.2	8.0	
New England	2.0	1.9	2.1	1.5	1.6	1.5	
Pacific	6.6	6.7	6.6	7.0	7.1	6.9	
South Atlantic	26.3	24.4	26.5	25.3	25.2	25.3	
West North Central	4.9	5.1	4.8	5.8	5.7	5.9	
West South Central	13.7	13.2	13.8	18.0	15.7	18.6	
Comorbid diagnoses (at hospital discharge), %							
Atrial fibrillation	17.6	27.4	16.5	26.5	32.0	25.0	< 0.0001
Cerebrovascular disease	5.4	19.7	3.7	8.6	22.0	5.0	< 0.0001
Chronic obstructive pulmonary disease	14.8	16.9	14.5	18.4	17.9	18.5	< 0.0001
Congestive heart failure	17.1	29.0	15.7	23.7	31.6	21.6	< 0.0001
Coronary artery disease	22.7	42.6	20.5	28.9	44.5	24.7	< 0.0001
Diabetes mellitus	40.9	47.5	40.2	50.7	53.0	50.0	< 0.0001
Hyperlipidemia	40.4	48.5	39.5	44.2	48.4	43.1	< 0.0001
Hypertension	67.4	77.5	66.3	73.8	78.2	72.7	< 0.0001
Inflammatory bowel disease	0.6	0.6	0.6	0.6	0.6	0.6	0.0345
Liver disease	6.3	7.6	6.2	8.9	9.9	8.7	< 0.0001
Obesity	28.6	28.2	28.6	36.6	33.8	37.3	< 0.0001
Peptic ulcer disease	0.8	1.6	0.7	1.6	2.3	1.4	< 0.0001
Peripheral vascular disease	3.9	8.2	3.4	5.2	9.1	4.1	< 0.0001
Renal disease	24.8	37.4	23.4	36.3	44.2	34.2	< 0.0001
Rheumatologic disease	2.4	2.6	2.4	2.6	2.5	2.6	0.0001
Any malignancy	4.4	5.7	4.3	4.9	5.2	4.9	< 0.0001

*COVID-19* coronavirus disease 2019, *ICU* intensive care unit, *SD* standard deviation, *US* United States

When comparing hospitalized patients with COVID-19 by ICU admission status (ICU versus [vs] non-ICU), ICU patients were more likely to be male (58.7% vs 51.5%) and have a comorbid diagnosis of renal disease (36.3% vs 24.8%), obesity (36.6% vs 28.6%), diabetes mellitus (50.7% vs 40.9%), atrial fibrillation (26.5% vs 17.6%), hypertension (73.8% vs 67.4%), congestive heart failure (23.7% vs 17.1%), or coronary artery disease (28.9% vs 22.7%) (*P* < 0.0001 for all; Table 1).

Demographic and clinical characteristics were also calculated for all hospitalized (ICU plus non-ICU) and ICU inpatients with COVID-19 by the presence or absence of a diagnosis of a thrombotic event (Table 1). Notably, among all hospitalized and ICU inpatients with COVID-19, the median first-measurement D-dimer values for patients with thrombotic events (2160.0 and 2290.0 ng/mL fibrinogen equivalent units [FEU], respectively) were higher than among those without thrombotic events (1000.0 and 1290.0 ng/mL FEU, respectively). Detailed laboratory data are in Supplementary Table 3.

### Thrombotic and bleeding events, mechanical ventilation, and death in matched pairs

**Fig. 1:**
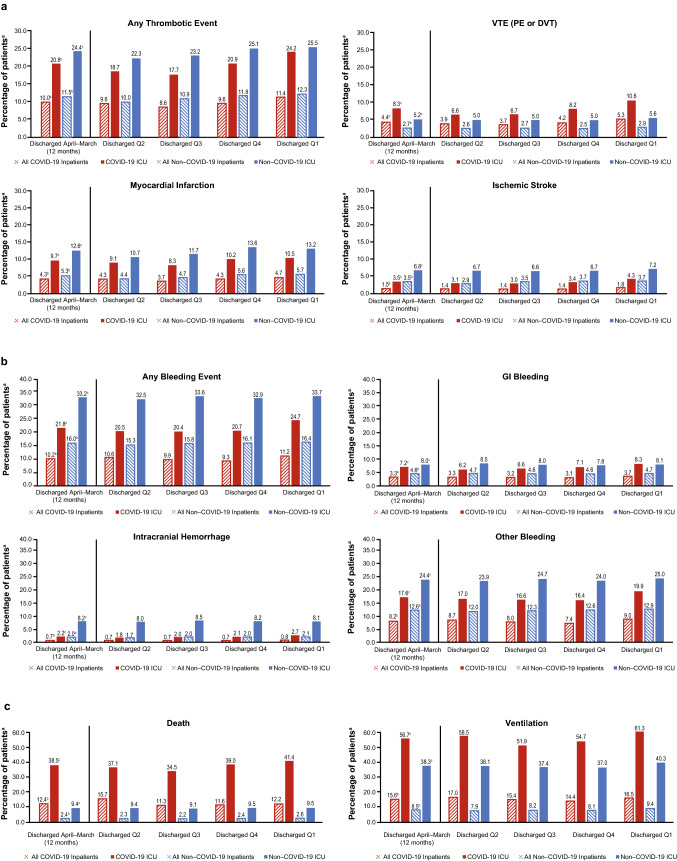
Thrombotic events (**a**), bleeding events (**b**), and mortality and mechanical ventilation (**c**) among matched pairs of hospitalized and ICU patients with and without COVID-19. ^a^Percentages are based on the number of patients in each cohort. Historical control and COVID-19 cohorts are matched for all hospitalized and ICU comparisons. The COVID-19 patient group was discharged between April 2020 and March 2021; the historical control group was discharged between April 2018 and March 2019. Hospitalized inpatients: discharged from April to March (12 months), N = 515,132; discharged in Q2, n = 81,598; discharged in Q3, n = 93,805; discharged in Q4, n = 180,019; discharged in Q1, n = 159,710. ICU patients: discharged from April–March (12 months), n = 86,197; discharged in Q2, n = 14,193; discharged in Q3, n = 17,730; discharged in Q4, n = 28,971; discharged in Q1 2021, n = 25,303. ^b^*P* < 0.0001 for the comparisons of all COVID-19 inpatients vs all non–COVID-19 inpatients. ^c^*P* < 0.0001 for the comparisons of COVID-19 ICU patients vs non–COVID-19 ICU patients. *COVID-19* coronavirus disease 2019, *DVT* deep vein thrombosis, *GI* gastrointestinal, *ICU* intensive care unit, *PE* pulmonary embolism, *Q* quarter, *Q1* January–March, *Q2* April–June, *Q3* July–September, *Q4* October–December, *VTE* venous thromboembolism

A total of 515,132 hospitalized (ICU plus non-ICU) and 86,197 ICU inpatients with COVID-19 were matched 1:1 with historical controls. Demographic and clinical characteristics for the matched pairs of COVID-19 and historical control inpatients are in Supplementary Table 4. Any thrombotic event was diagnosed in 10.0% of hospitalized and 20.8% of ICU inpatients with COVID-19 vs 11.5% and 24.4% of historical controls, respectively (Fig. 1a). When evaluating specific thrombotic events, a higher rate of VTE and lower rate of ischemic stroke and myocardial infarction were observed among hospitalized and ICU inpatients with COVID-19 vs their respective historical controls (*P* < 0.0001 for all; Fig. 1a). Among ICU inpatients with COVID-19, VTE increased from 6.6% in quarter 2 (April–June) 2020 to 10.6% in quarter 1 (January–March) 2021. For detailed data on the types of thrombotic events in patients with COVID-19 vs historical controls, see Supplementary Table 5 (hospitalized patients) and Supplementary Table 6 (ICU patients).

In the matched pairs, bleeding events were observed in 10.2% of hospitalized (ICU plus non-ICU) and 21.8% of ICU inpatients with COVID-19 vs 16.0% (*P* < 0.0001) and 33.2% (*P* < 0.0001) for historical controls, respectively (Fig. 1b). Death (hospitalized: 12.4% vs 2.4%; ICU: 38.5% vs 9.4%) and mechanical ventilation were increased among hospitalized and ICU inpatients with COVID-19 vs historical controls (*P* < 0.0001 for all; Fig. 1c). Detailed information on thrombotic and bleeding events, death, and mechanical ventilation by discharge quarter are in Fig. 1.

### Anticoagulant use

**Fig. 2:**
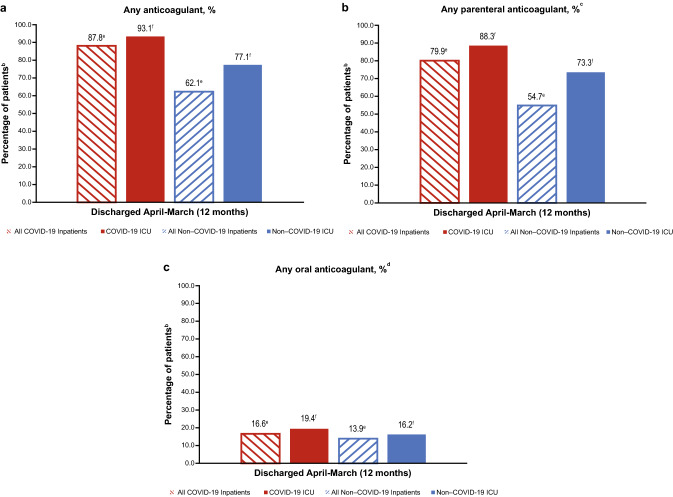
Anticoagulant use^a^ among matched pairs of hospitalized and ICU patients with and without COVID-19. ^a^Patients may have received more than one anticoagulant and were counted for each anticoagulant they received for ≥ 1 day. ^b^Percentages are based on the number of patients in each cohort. Historical control and COVID-19 cohorts are matched for all hospitalized and ICU comparisons. The COVID-19 patient group was discharged between April 2020–March 2021; the historical control group was discharged between April 2018–March 2019. Hospitalized inpatients: discharged from April–March (12 months), N = 515,132. ICU patients: discharged from April–March (12 months), n = 86,197. ^c^Parenteral anticoagulants included LMWH-enoxaparin or dalteparin, heparin, and fondaparinux. ^d^Oral anticoagulants included warfarin, apixaban, dabigatran, edoxaban, and rivaroxaban. ^e^*P* < 0.0001 for the comparison of all COVID-19 inpatients vs all non–COVID-19 inpatients. ^f^*P* < 0.0001 for the comparison of COVID-19 ICU patients vs non–COVID-19 ICU patents. *COVID-19* coronavirus disease 2019, *ICU* intensive care unit, *LMWH* low-molecular-weight heparin

For all matched inpatients with COVID-19 (ICU plus non-ICU), 87.8% used any anticoagulant for ≥ 1 day; 79.9% and 16.6% of patients used parenteral anticoagulants and OACs, respectively (Fig. 2). Among matched ICU inpatients with COVID-19, 93.1% used any anticoagulants for ≥ 1 day; 88.3% and 19.4% of patients used parenteral anticoagulants and OACs, respectively. Historical controls used any anticoagulant, parenteral anticoagulants, and OACs at a lower rate than matched COVID-19 inpatients (all *P* < 0.0001, Fig. 2).

When all hospitalized (ICU plus non-ICU) inpatients with COVID-19 were stratified by presence or absence of a thrombotic event, 94.6% of patients with a thrombotic event used any anticoagulant and 88.7% used parenteral anticoagulants; rates in patients without thrombotic events were slightly lower (Table 2). OAC use was higher for all hospitalized and ICU inpatients with COVID-19 who had a thrombotic event vs those without a thrombotic event (all hospitalized: 36.8% vs 14.3%; ICU: 29.6% vs 16.0%; all *P* < 0.0001). Detailed information on anticoagulant use by dosage is in Supplementary Table 7; additional data on anticoagulant use by quarter for matched pairs of patients with and without COVID-19 are in Supplementary Table 8 (hospitalized patients) and Supplementary Table 9 (ICU patients).

**Table 2:** Hospitalization outcomes and use of anticoagulants among hospitalized patients with COVID-19 overall and by presence or absence of a diagnosis of a thrombotic event

	All COVID-19 Inpatients			All COVID-19 ICU Inpatients		
	Total N = 546,656	Thrombotic event n = 56,015	No thrombotic event n = 490,641	Total n = 110,111	Thrombotic event n = 23,461	No thrombotic event n = 86,650
	*Hospitalization outcomes*					
Hospital stay, days, mean (SD)	8.2 (9.3)	12.6 (13.5)	7.7 (8.5)	15.1 (13.7)	17.8 (16.4)	14.4 (12.7)
Hospital stay, days, median (Quartile 1, Quartile 3)	5.0 (3.0, 10.0)	8.0 (4.0, 16.0)	5.0 (3.0, 9.0)	12.0 (6.0, 20.0)	14.0 (7.0, 24.0)	11.0 (6.0, 19.0)
ICU admission, %	20.1	41.9^d^	17.7^d^	100.0	100.0^d^	100.0^d^
ICU stay, days, mean (SD)	9.1 (10.7)	11.1 (12.8)	8.6 (10.0)	9.1 (10.7)	11.1 (12.8)	8.6 (10.0)
ICU stay, days, median (Quartile 1, Quartile 3)	5.0 (2.0, 12.0)	7.0 (2.0, 15.0)	5.0 (2.0, 11.0)	5.0 (2.0, 12.0)	7.0 (2.0, 15.0)	5.0 (2.0, 11.0)
Mechanical ventilation, %	16.6	37.6^d^	14.2^d^	59.4	70.9^d^	56.3^d^
Mortality, %	12.7	29.4^d^	10.8^d^	38.6	48.7^d^	35.8^d^
	*Anticoagulant use*^a^					
Any anticoagulant, %	88.0	94.6^d^	87.3^d^	93.5	95.4^d^	92.9^d^
Any parenteral anticoagulant^b^, %	80.4	88.7^d^	79.5^d^	89.3	93.0^d^	88.4^d^
Any oral anticoagulant^c^, %	16.6	36.8^d^	14.3^d^	18.9	29.6^d^	16.0^d^

*COVID-19* coronavirus disease 2019, *ICU* intensive care unit, *LMWH* low-molecular-weight heparin, *SD* standard deviation

^a^Patients may have received more than one anticoagulant and were counted for each anticoagulant they received for ≥ 1 day

^b^Parenteral anticoagulants included LMWH-enoxaparin or dalteparin, heparin, and fondaparinux

^c^Oral anticoagulants included warfarin, apixaban, dabigatran, edoxaban, and rivaroxaban

^d^*P* < 0.0001 for the comparison of thrombotic event vs no thrombotic event

### Hospitalization outcomes by presence or absence of a diagnosis of a thrombotic event

Among all hospitalized (ICU plus non-ICU) and ICU inpatients with COVID-19, patients with a thrombotic event had longer median hospital stays than those without a thrombotic event (8 vs 5 days for all hospitalized; 14 vs 11 days for ICU; Table 2). Additionally, a higher proportion of inpatients with COVID-19 who had a thrombotic event were admitted to ICU (41.9% vs 17.7%; P < 0.0001), needed mechanical ventilation (37.6% vs 14.2%; *P* < 0.0001) or died (29.4% vs 10.8%; *P* < 0.0001) vs those without a thrombotic event. All ICU inpatients with COVID-19 and a thrombotic event were also more likely to need mechanical ventilation (70.9% vs 56.3%; *P* < 0.0001) or die (48.7% vs 35.8%; *P* < 0.0001) than those without a thrombotic event.

## Discussion

Of the 515,132 matched, hospitalized patients with COVID-19 in this US national database, 10.0% were diagnosed with any thrombotic events (20.8% for ICU patients), and 10.2% were diagnosed with any bleeding events (21.8% for ICU patients). Both thrombotic and bleeding event rates were higher in historical controls vs inpatients with COVID-19, regardless of ICU admission status (Fig. 1). However, a higher rate of VTE and mortality was observed in hospitalized and ICU patients with COVID-19 vs historical controls (Fig. 1). Most hospitalized and ICU inpatients with COVID-19 used anticoagulants for ≥ 1 day. Despite common use of anticoagulants in patients with COVID-19, patients with thrombotic events had longer hospital stay and higher mortality than patients without thrombotic events.

There is considerable variation in VTE rates in patients with COVID-19 reported in the literature, ranging from 3–23% in non-ICU patients and 8–69% in ICU patients with COVID-19 [6]. This variation may be due to differences in the classification and adjudication of events, routine image screening, and thromboprophylaxis and treatment protocols. Our VTE event rate of 4.4% in hospitalized patients with COVID-19 and 8.3% in ICU patients with COVID-19 during the period of April 2020–March 2021 was in the lower range of what have been reported in the literature. While we observed an increased VTE event rate among ICU patients with COVID-19 in later vs earlier observation periods (April–June 2020 vs January–March 2021), the VTE event rate for ICU patients was still relatively low compared with many other studies [6].

There is a lack of bleeding event rate data among hospitalized patients with COVID-19. In this study, we found 10.2% of hospitalized patients and 21.8% of ICU patients with COVID-19 had any bleeding event. Both rates were lower than that of the respective historical controls, although patients with COVID-19 were more likely to receive anticoagulants vs historical controls. It is important to note that historical control patients may have had different comorbidities and received different treatments (e.g., they may have received surgeries which led to a higher rate of bleeding). Despite being lower than those of the historical controls, the bleeding event rates observed in our study were higher than another retrospective analysis which reported 4.8% of hospitalized and 7.6% of critically ill patients with COVID-19 who had a bleeding event [5]. Further research is needed to better understand the risk of bleeding among hospitalized patients with COVID-19.

Notably, thrombotic events had a large impact on hospitalized patients with COVID-19. Our study found that mortality rates tripled in hospitalized patients with COVID-19 who had a thrombotic event when compared with those who did not have a thrombotic event (29.4% vs 10.8%), despite anticoagulant use in the majority of patients with a thrombotic event. These findings align with results of a recent meta-analysis that showed thrombotic events in patients with COVID-19 were associated with a higher risk of death [6], and suggest that more efforts are needed to identify optimal types and dosages of anticoagulants, as well as approaches beyond anticoagulants, to reduce mortality among hospitalized patients with COVID-19 who have a thrombotic event.

Our findings that (1) increased rates of anticoagulant use did not prevent a high mortality rate in patients with COVID-19 who had a thrombotic event and (2) the higher rates of bleeding we found in comparison to the literature, raise the question of whether intense anticoagulation in critically ill patients with COVID-19 is warranted. The recently completed INSPIRATION randomized controlled trial (RCT) found that ICU patients with COVID-19 treated prophylactically with intermediate-dose anticoagulants did not have significant differences in VTE or mortality rates compared with patients treated with standard-dose anticoagulants [13]. Another RCT reported that intermediate-dose enoxaparin was not more effective in preventing death or thrombosis than standard-dose enoxaparin in hospitalized patients with COVID-19 [14], and the ACTION RCT found that anticoagulation with rivaroxaban or enoxaparin, followed by rivaroxaban for 30 days, did not improve clinical outcomes and led to increased bleeding when compared with prophylactic anticoagulation [15]. Observational studies have shown inadequate anticoagulation and limited benefit of prophylactic anticoagulation on mortality rates in patients with COVID-19 [16–19].

Another area of debate is whether antiplatelet therapies should be used in combination with anticoagulants for the treatment of patients with COVID-19. Results of the ACTIVE-4a trial, which evaluated non-critically ill, hospitalized patients with COVID-19, found that the addition of a P2Y12 inhibitor to a therapeutic dose of heparin did not improve survival [20]. Other ongoing ACTIV-4 studies aim to provide more insights about the safety and efficacy of antithrombotic strategies for hospitalized patients with COVID-19 [21].

Despite using a large national database, this study has limitations. Thrombotic events were identified based on discharge diagnoses, and the difficulty in conducting radiologic imaging on patients with COVID-19 may have led to under-reporting of events that require imaging confirmation [22]. Whether anticoagulants were used for prophylaxis or treatment could not be determined due to a lack of available data reported in the administrative claims database, and the duration of anticoagulant use was not evaluated. Bleeding severity was not assessed, and the relationship between bleeding events and anticoagulant use, procedures, or other factors could not be determined. Although we have matched the historical controls with the COVID-19 cohort on several factors, there may be other differences between the two cohorts, such as comorbidities and treatments. Also, even though we did show an increase in mortality rates in hospitalized patients with COVID-19 who had a thrombotic event when compared with those who did not, we were limited in our ability to fully analyze correlates of mortality. Finally, the age, race, and comorbidity profile of patients with COVID-19 included in this study varied compared with data collected by the Centers for Disease Control and Prevention [23], and results of this study cannot be generalized to patients outside of this dataset.

## Conclusions

In this large national study, rates of VTE and mortality were higher in hospitalized and ICU patients with COVID-19 than in historical controls. Although anticoagulants were commonly prescribed among hospitalized patients with COVID-19 who had a thrombotic event, these patients experienced worse outcomes, including higher mortality, than those without thrombotic events. More studies investigating the optimal types and dosages of thromboprophylactic agents (e.g. anticoagulants, statins) in patients with COVID-19 are needed.

## Supplementary Information

Below is the link to the electronic supplementary material.

Supplementary file1 (DOCX 83 kb)

## Data Availability

Data queries should be addressed to Premier Inc., which is an independent third-party with rights to data availability in the Premier Healthcare Database. These data are proprietary, and the authors do not have permission to disseminate them; however, they can be obtained from the vendor at a cost. Please contact the vendor for additional information.
