# Supplementary material for: Thrombotic and bleeding events, mortality, and anticoagulant use among 546,656 hospitalized patients with COVID-19 in the United States: a retrospective cohort study
Source: J Thromb Thrombolysis. 2022 Apr 30;53(4):766–76. doi: 10.1007/s11239-022-02644-2 (PMC9055213; doi:10.1007/s11239-022-02644-2)
Supplement: Supplementary file 1 — Supplementary file1 (DOCX 83 kb) [file 11239_2022_2644_MOESM1_ESM.docx]

**Title:** Thrombotic and Bleeding Events, Mortality, and Anticoagulant Use Among 546,656 Hospitalized Patients With COVID-19 in the United States: A Retrospective Cohort Study

**Authors:** Steve Deitelzweig, MD^1^; Xuemei Luo, PhD^2^; Jennifer L. Nguyen, ScD, MPH^3^; Deepa Malhotra, MS, MBA^3^; Birol Emir, PhD^3^; Cristina Russ, MD^3^; Xiaoyan Li, PhD^4^; Theodore C. Lee, MD^3^; Mauricio Ferri, MD^4^; Danny Wiederkehr, MPH^3^; Maya Reimbaeva, MS^2^; Geoffrey D. Barnes, MD, MSc^5^*; Gregory Piazza, MD, MS^6^**^*^**

*GD Barnes and G Piazza contributed equally

**Author affiliations:**

^1^Ochsner Clinic Foundation, Department of Hospital Medicine and The University of Queensland School of Medicine, Ochsner Clinical School, New Orleans, LA, USA; ^2^Pfizer Inc., Groton, CT, USA; ^3^Pfizer Inc., New York, NY, USA; ^4^Bristol Myers Squibb Company, Lawrenceville, NJ, USA; ^5^Frankel Cardiovascular Center, University of Michigan, Ann Arbor, MI, USA; ^6^Brigham and Women’s Hospital, Boston, MA, USA

**Corresponding author:**

Steve Deitelzweig, MD

Ochsner Medical Center

1514 Jefferson Hwy

New Orleans, LA 70121

**Telephone:** 504-842-5766

**Fax:** 504-842-6617

**Email:** [sdeitelzweig@ochsner.org](mailto:sdeitelzweig@ochsner.org)

**Supplementary Table 1** ICD-10-CM diagnostic codes used to identify thrombotic events

**Supplementary Table 2** ICD-10-CM and PCS diagnostic codes used to identify bleeding events

**Supplementary Table 3** Median laboratory values among hospitalized and ICU patients with COVID-19: Overall and by presence or absence of a diagnosis of a thrombotic event

**Supplementary Table 4** Demographic and clinical characteristics of matched pairs of hospitalized and ICU patients with and without COVID-19

**Supplementary Table 5** Thrombotic events and other severe outcomes in matched pairs of hospitalized patients with and without COVID-19 by discharge quarter^a^

**Supplementary Table 6** Thrombotic events and shock in matched pairs of hospitalized patients admitted to the ICU with and without COVID-19 by discharge quarter^a^

**Supplementary Table 7** Types of anticoagulants used among hospitalized patients with COVID-19 overall and by presence or absence of a diagnosis of a thrombotic event

**Supplementary Table 8** Use of anticoagulants by quarter among matched pairs of hospitalized patients with and without COVID-19^a^

**Supplementary Table 9** Use of anticoagulants by quarter among matched pairs of hospitalized patients admitted to the ICU with and without COVID-19^a^

**Supplementary Table 1** ICD-10-CM diagnostic codes used to identify thrombotic events

| **Conditions** | **ICD-10-CM Code(s)** |
| --- | --- |
| Acute coronary syndrome | I24.9 |
| Acute myocardial infarction | I21 |
| Arterial thromboembolism | I74.01, I74.09, I74.10, I74.11, I74.19, I74.2, I74.3, I74.4, I74.5, I74.8, I74.9 |
| DIC | D65 |
| DVT | |
| Acute lower extremity DVT | I82.40x, I82.41x, I82.42x, I82.43x, I82.44x, I82.49x, I82.4Yx, I82.4Zx |
| Lower extremity phlebitis | I80.1x, I80.20x, I80.21x, I80.22x, I80.23x, I80.29x, I80.3, I80.8, I80.9 |
| Superior vena cava thrombosis | I82.210, I82.220, I82.290, I82.3 |
| Upper extremity DVT | I82.60x, I82.62x, I82.A1x, I82.B1x, I82.C1x |
| Ischemic stroke | I63.00, I63.01x, I63.02, I63.03x, I63.09, I63.10, I63.11x, I63.12, I63.13x, I63.19, I63.20, I63.21x, I63.22, I63.23x, I63.29, I63.30, I63.31x, I63.32x, I63.33x, I63.34x, I63.39, I63.40, I63.41x, I63.42x, I63.43x, I63.44x, I63.49, I63.50, I63.51x, I63.52x, I63.53x, I63.54x, I63.59, I63.013, I63.033, I63.113, I63.133, I67.82, I63.6, I63.8, I63.9 |
| Pulmonary embolism | I26.02, I26.09, I26.92, I26.99 |

DIC, disseminated intravascular coagulation; DVT, deep vein thrombosis; ICD-10-CM, International Classification of Diseases, Tenth Revision, Clinical Modification.

**Supplementary Table 2** ICD-10-CM and PCS diagnostic codes used to identify bleeding events

| **Conditions** | **ICD-10-CM and PCS Code(s)** |
| --- | --- |
| Gastrointestinal bleeding | **ICD-10-CM Codes:** I8501, I8511, K2211, K226, K250, K252, K254, K256, K260, K262, K264, K266, K270, K272, K274, K276, K280, K282, K284, K286, K2901, K2921, K2931, K2941, K2951, K2961, K2971, K2981, K2991, K31811, K3182, K5521, K5701, K5711, K5713, K5721, K5731, K5733, K5741, K5751, K5753, K5781, K5791, K5793, K625, K6381, K661, K920, K921, K922, K9161, K9162, K91840, K91841  **PCS Codes**: no codes |
| Intracranial hemorrhage | **ICD-10-CM Codes:** I6000, I6001, I6002, I6010, I6011, I6012, I602, I6030, I6031, I6032, I604, I6050, I6051, I6052, I606, I607, I608, I609, I610, I611, I612, I613, I614, I615, I616, I618, I619, I6200, I6201, I6202, I6203, I621, I629, S06340A, S06341A, S06342A, S06343A, S06344A, S06345A, S06346A, S06347A, S06348A, S06349A, S06350A, S06351A, S06352A, S06353A, S06354A, S06355A, S06356A, S06357A, S06358A, S06359A, S06360A, S06361A, S06362A, S06363A, S06364A, S06365A, S06366A, S06367A, S06368A, S06369A, S064X0A, S064X1A, S064X2A, S064X3A, S064X4A, S064X5A, S064X6A, S064X7A, S064X8A, S064X9A, S065X0A, S065X1A, S065X2A, S065X3A, S065X4A, S065X5A, S065X6A, S065X7A, S065X8A, S065X9A, S066X0A, S066X1A, S066X2A, S066X3A, S066X4A, S066X5A, S066X6A, S066X7A, S066X8A, S066X9A  **PCS Codes**: no codes |
| Other hemorrhage | **ICD-10 CM Codes:** D62, D7801, D7802, D7821, D7822, E3601, E3602, E89810, E89811, G9731, G9732, G9751, G9752, H05231, H05232, H05233, H05239, H1130, H1131, H1132, H1133, H2100, H2101, H2102, H2103, H31301, H31302, H31303, H31309, H31311, H31312, H31313, H31319, H31411, H31412, H31413, H31419, H3560, H3561, H3562, H3563, H35731, H35732, H35733, H35739, H4310, H4311, H4312, H4313, H44811, H44812, H44813, H44819, H47021, H47022, H47023, H47029, H59111, H59112, H59113, H59119, H59121, H59122, H59123, H59129, H59311, H59312, H59313, H59319, H59321, H59322, H59323, H59329, H9521, H9522, H9541, H9542, I312, I97410, I97411, I97418, I9742, I97610, I97611, I97618, I97620, J9561, J9562, J95830, J95831, L7601, L7602, L7621, L7622, M2500, M25011, M25012, M25019, M25021, M25022, M25029, M25031, M25032, M25039, M25041, M25042, M25049, M25051, M25052, M25059, M25061, M25062, M25069, M25071, M25072, M25073, M25074, M25075, M25076, M2508, M96810, M96811, M96830, M96831, N421, N857, N897, N920, N923, N930, N938, N939, N9961, N9962, N99820, N99821, R040, R041, R042, R0489, R049, R233, R310, R319, R58, T792XXA  **PCS Codes:** 30230N1, 30230P1, 30233N1, 30233P1, 30240N1, 30240P1, 30243N1, 30243P1, 30250N1, 30250P1, 30253N1, 30253P1, 30260N1, 30260P1, 30263N1, 30263P1 |

ICD-10-CM, International Classification of Diseases, Tenth Revision, Clinical Modification; PCS, procedure coding system.

**Supplementary Table 3** Median laboratory values among hospitalized and ICU patients with COVID-19: Overall and by presence or absence of a diagnosis of a thrombotic event

| **Median Laboratory Values** | **All COVID-19 Inpatients** | | | **All COVID-19 ICU Inpatients** | | |
| --- | --- | --- | --- | --- | --- | --- |
|  | **Total**  **N = 546,656** | **Thrombotic Event**  **n = 56,015** | **No Thrombotic Event**  **n = 490,641** | **Total**  **n = 110,111** | **Thrombotic Event**  **n = 23,461** | **No Thrombotic Event**  **n = 86,650** |
| D-dimer, n | 72,441.0 | 7,682 | 64,759 | 17,473 | 3,715 | 13,758 |
| First measurement  (quartile 1, quartile 3), ng/mL FEU | 1,060  (610.0, 2,074.0) | 2,160.0  (1,020.0 5,870.0) | 1,000  (586.0, 1,870.0) | 1,428.0  (774.0, 3,300.0) | 2,290.0  (1,080.0, 6,594.0) | 1,290.0  (721.0, 2,670.0) |
| Maximum measurement, ng/mL FEU | 1,400 | 4,121 | 1,270.0 | 3,390.0 | 6,951.0 | 2,790 |
| Prothrombin time, n | 73,087 | 10,335 | 62,752 | 20,954 | 5,139 | 15,815 |
| First measurement  (quartile 1, quartile 3), s | 14.0  (12.4, 14.9) | 14.0  (12.7, 15.8) | 14.0  (12.3, 14.8) | 14.0  (12.5, 15.5) | 14.0  (12.8, 16.1) | 14.0  (12.4, 15.3) |
| Partial thromboplastin time, n | 53,075 | 9,108 | 43,967 | 16,608 | 4,624 | 11,984 |
| First measurement  (quartile 1, quartile 3), s | 32.0  (28.2, 36.1) | 32.0  (28.0, 38.0) | 32.0  (28.3, 36.0) | 32.0  (28.1, 37.5) | 32.0  (28.0, 38.4) | 32.0  (28.3, 37.1) |
| Fibrinogen, n | 24,113 | 3,824 | 20,289 | 9,486 | 2,540 | 6,946 |
| First measurement  (quartile 1, quartile 3), mg/dL | 536.0  (419.0, 662.0) | 489.0  (346.0, 633.5) | 543.0  (430.0, 667.0) | 538.0  (396.0, 676.0) | 490.0  (332.0, 640.0) | 555.0  (420.0, 686.0) |
| Maximum measurement, mg/dL | 567.0 | 537.0 | 571.0 | 592.0 | 550.0 | 600.0 |
| Minimum measurement, mg/dL | 465.0 | 395.0 | 476.0 | 429.0 | 372.0 | 447.0 |
| Platelet count, n | 124,152 | 12,714 | 111,438 | 27,082 | 5,735 | 21,347 |
| First measurement  (quartile 1, quartile 3), 1000/µL | 210.0  (162.0, 273.0) | 217.0  (162.0, 290.0) | 210.0  (162.0, 271.0) | 212.0  (160.0, 279.0) | 218.0  (161.0, 290.0) | 210.0  (159.0, 276.0) |
| Maximum measurement, 1000/µL | 281.0 | 295.0 | 280.0 | 315.0 | 313.0 | 315.0 |
| Minimum measurement, 1000/µL | 184.0 | 166.0 | 186.0 | 157.0 | 144.0 | 160.0 |
| Lymphocyte count, n | 112,361 | 11,506 | 100,855 | 24,996 | 5,201 | 19,795 |
| First measurement  (quartile 1, quartile 3), x 1000/µL | 1.0  (0.6, 1.38) | 1.0  (0.6, 1.4) | 1.0  (0.6, 1.4) | 1.0  (0.5, 1.2) | 1.0  (0.5, 1.3) | 1.0  (0.6, 1.2) |
| Maximum measurement, x1000/µL | 1.0 | 1.0 | 1.0 | 1.0 | 2.0 | 1.0 |
| Minimum measurement, x1000/µL | 1.0 | 1.0 | 1.0 | 0 | 0 | 0 |

COVID-19, coronavirus disease 2019; FEU, fibrinogen equivalent units; ICU, intensive care unit.

**Supplementary Table 4** Demographic and clinical characteristics of matched pairs of hospitalized and ICU patients with and without COVID-19

|  | **Matched Pairs of Inpatients** | | **Matched Pairs of ICU Inpatients** | |
| --- | --- | --- | --- | --- |
| **Characteristics** | **COVID-19 Inpatients**  **N = 515,132** | **Historical Control  Non–COVID-19 Inpatients**  **N = 515,132** | **COVID-19 ICU Inpatients**  **N = 86,197** | **Historical Control  Non–COVID-19 ICU Inpatients**  **N = 86,197** |
| Age, years, mean (SD) | 63.3 (18.0) | 63.3 (18.0) | 65.4 (15.5) | 65.4 (15.5) |
| Age group, % |  |  |  |  |
| < 18 years | 0.9 | 0.9 | 0.8 | 0.8 |
| 18–39 years | 11.2 | 11.2 | 6.0 | 6.0 |
| 40–59 years | 24.7 | 24.7 | 23.0 | 23.0 |
| 60–79 years | 43.2 | 43.2 | 52.5 | 52.5 |
| ≥ 80 years | 20.1 | 20.1 | 17.7 | 17.7 |
| Sex, % |  |  |  |  |
| Male | 50.9 | 50.9 | 58.4 | 58.4 |
| Female | 49.1 | 49.1 | 41.6 | 41.6 |
| Unknown | 0 | 0 | 0 | 0 |
| Race, % |  |  |  |  |
| White | 66.9 | 66.9 | 73.3 | 73.3 |
| Black | 17.7 | 17.7 | 17.1 | 17.1 |
| Asian | 2.0 | 2.0 | 1.1 | 1.1 |
| Other | 10.4 | 10.4 | 7.3 | 7.3 |
| Unknown | 3.0 | 3.0 | 1.2 | 1.2 |
| Ethnicity, % |  |  |  |  |
| Hispanic | 15.6 | 15.6 | 12.2 | 12.2 |
| Not Hispanic | 68.8 | 68.8 | 73.4 | 73.4 |
| Unknown | 15.6 | 15.6 | 14.4 | 14.4 |
| US Census division, % |  |  |  |  |
| East North Central | 15.7 | 15.7 | 17.3 | 17.3 |
| East South Central | 7.4 | 7.4 | 7.3 | 7.3 |
| Middle Atlantic | 15.8 | 15.8 | 10.8 | 10.8 |
| Mountain | 7.4 | 7.4 | 6.6 | 6.6 |
| New England | 1.9 | 1.9 | 1.1 | 1.1 |
| Pacific | 6.4 | 6.4 | 5.4 | 5.4 |
| South Atlantic | 26.9 | 26.9 | 27.4 | 27.4 |
| West North Central | 4.7 | 4.7 | 5.6 | 5.6 |
| West South Central | 13.8 | 13.8 | 18.4 | 18.4 |
| Comorbid diagnoses  (at hospital discharge), % |  |  |  |  |
| Atrial fibrillation | 17.7 | 17.9 | 27.9 | 27.1 |
| Cerebrovascular disease | 5.3 | 8.8 | 8.5 | 16.5 |
| Chronic obstructive pulmonary disease | 15.1 | 16.7 | 20.2 | 23.1 |
| Congestive heart failure | 17.2 | 19.4 | 24.8 | 29.1 |
| Coronary artery disease | 23.0 | 27.0 | 30.7 | 39.5 |
| Diabetes mellitus | 40.5 | 32.2 | 49.9 | 38.5 |
| Hyperlipidemia | 40.7 | 36.9 | 46.0 | 43.1 |
| Hypertension | 67.6 | 66.3 | 75.5 | 74.9 |
| Inflammatory bowel disease | 0.6 | 0.9 | 0.6 | 0.7 |
| Liver disease | 6.2 | 7.2 | 8.5 | 10.0 |
| Obesity | 28.5 | 18.4 | 36.1 | 20.7 |
| Peptic ulcer disease | 0.7 | 1.7 | 1.4 | 2.9 |
| Peripheral vascular disease | 3.9 | 6.2 | 5.5 | 10.3 |
| Renal disease | 24.6 | 20.9 | 36.3 | 28.4 |
| Rheumatologic disease | 2.4 | 2.5 | 2.8 | 2.4 |
| Any malignancy | 4.5 | 9.3 | 5.2 | 10.9 |

COVID-19, coronavirus disease 2019; ICU, intensive care unit; SD, standard deviation; US, United States.

**Supplementary Table 5** Thrombotic events and other severe outcomes in matched pairs of hospitalized patients with and without COVID-19 by discharge quarter^a^

|  | **Matched-pairs Comparison** | | | | | | | | | |
| --- | --- | --- | --- | --- | --- | --- | --- | --- | --- | --- |
| **Outcomes** | **COVID-19 Inpatients** | | | | | **Historical Controls: Non–COVID-19 Inpatients** | | | | |
|  | **Discharged  Q2 2020**  **n = 81,598** | **Discharged Q3 2020**  **n = 93,805** | **Discharged Q4 2020**  **n = 180,019** | **Discharged Q1 2021**  **n = 159,710** | **Total  Discharged  Apr 2020–Mar 2021**  **N = 515,132** | **Discharged  Q2 2018**  **n = 81,598** | **Discharged  Q3 2018**  **n = 93,805** | **Discharged  Q4 2018**  **n = 180,019** | **Discharged  Q1 2019**  **n = 159,710** | **Total  Discharged  Apr 2018–Mar 2019**  **N = 515,132** |
| Shock, % | 0.8 | 0.8 | 0.7 | 0.9 | 0.8^b^ | 0.2 | 0.3 | 0.3 | 0.3 | 0.3^b^ |
| ICU admission, % | 20.7 | 22.0 | 18.0 | 18.2 | 19.2^b^ | 13.5 | 16.1 | 15.8 | 16.4 | 15.7^b^ |
| Any thrombotic event, % | 9.6 | 8.6 | 9.7 | 11.4 | 10.0^b^ | 10.0 | 10.9 | 11.8 | 12.3 | 11.5^b^ |
| VTE (PE or DVT) | 3.9 | 3.7 | 4.2 | 5.3 | 4.4^b^ | 2.6 | 2.7 | 2.5 | 2.9 | 2.7^b^ |
| PE | 2.2 | 2.2 | 2.7 | 3.3 | 2.7^b^ | 1.2 | 1.3 | 1.3 | 1.5 | 1.4^b^ |
| DVT | 2.2 | 1.9 | 2.0 | 2.7 | 2.2^b^ | 1.8 | 1.8 | 1.7 | 2.0 | 1.8^b^ |
| Lower-extremity phlebitis | 0.2 | 0.1 | 0.1 | 0.2 | 0.1^b^ | 0.2 | 0.2 | 0.2 | 0.2 | 0.2^b^ |
| Lower-extremity DVT | 1.6 | 1.4 | 1.5 | 2.1 | 1.7^b^ | 1.2 | 1.3 | 1.2 | 1.4 | 1.3^b^ |
| Superior vena cava thrombosis | 0 | 0 | 0 | 0 | 0^b^ | 0.1 | 0.1 | 0.1 | 0.1 | 0.1^b^ |
| Upper-extremity DVT | 0.4 | 0.4 | 0.3 | 0.5 | 0.4^c^ | 0.4 | 0.4 | 0.3 | 0.4 | 0.4^c^ |
| DIC | 0.4 | 0.3 | 0.2 | 0.3 | 0.3^b^ | 0.1 | 0.2 | 0.1 | 0.2 | 0.2^b^ |
| Arterial thromboembolism | 0.3 | 0.2 | 0.2 | 0.3 | 0.2^b^ | 0.3 | 0.3 | 0.3 | 0.3 | 0.3^b^ |
| Ischemic stroke | 1.4 | 1.4 | 1.4 | 1.8 | 1.5^b^ | 2.9 | 3.5 | 3.7 | 3.7 | 3.5^b^ |
| Myocardial infarction | 4.3 | 3.7 | 4.3 | 4.7 | 4.3^b^ | 4.4 | 4.7 | 5.6 | 5.7 | 5.3^b^ |
| Acute coronary syndrome | 0.1 | 0.1 | 0.1 | 0.1 | 0.1^b^ | 0.2 | 0.2 | 0.3 | 0.2 | 0.2^b^ |

^a^Admissions were limited to April 2018 or later for historical control patients and April 2020 or later for COVID-19 inpatients.

^b^*P*<0.0001 for the matched comparisons of all COVID-19 inpatients vs all non-COVID-19 inpatients.

^c^*P*=0.0002 for the matched comparisons of all COVID-19 inpatients vs all non-COVID-19 inpatients.

Apr, April; COVID-19, coronavirus disease 2019; DIC, disseminated intravascular coagulation; DVT, deep vein thrombosis; ICU, intensive care unit; Mar, March; PE, pulmonary embolism; Q, quarter; Q1, January–March; Q2, April–June; Q3, July–September; Q4, October–December; VTE, venous thromboembolism.

**Supplementary Table 6** Thrombotic events and shock in matched pairs of hospitalized patients admitted to the ICU with and without COVID-19 by discharge quarter^a^

|  | **Matched-pairs Comparison** | | | | | | | | | |
| --- | --- | --- | --- | --- | --- | --- | --- | --- | --- | --- |
| **Outcomes** | **COVID-19 ICU Inpatients** | | | | | **Historical Controls: Non–COVID-19 ICU Inpatients** | | | | |
|  | **Discharged  Q2 2020**  **n = 14,193** | **Discharged Q3 2020**  **n = 17,730** | **Discharged Q4 2020**  **n = 28,971** | **Discharged Q1 2021**  **n = 25,303** | **Total  Discharged  Apr 2020–Mar 2021**  **N = 86,197** | **Discharged  Q2 2018**  **n = 14,193** | **Discharged  Q3 2018**  **n = 17,730** | **Discharged  Q4 2018**  **n = 28,971** | **Discharged  Q1 2019**  **n = 25,303** | **Total  Discharged  Apr 2018–Mar 2019**  **N = 86,197** |
| Shock, % | 2.8 | 2.9 | 3.0 | 3.8 | 3.2^b^ | 1.2 | 1.4 | 1.1 | 1.4 | 1.3^b^ |
| Any thrombotic event, % | 18.7 | 17.7 | 20.9 | 24.2 | 20.8^b^ | 22.3 | 23.2 | 25.1 | 25.5 | 24.4^b^ |
| VTE (PE or DVT) | 6.6 | 6.7 | 8.2 | 10.6 | 8.3^b^ | 5.0 | 5.0 | 5.0 | 5.6 | 5.2^b^ |
| PE | 3.0 | 3.5 | 4.6 | 5.6 | 4.4^b^ | 2.3 | 2.4 | 2.5 | 2.6 | 2.4^b^ |
| DVT | 4.1 | 3.9 | 4.7 | 6.4 | 4.9^b^ | 3.5 | 3.7 | 3.7 | 4.1 | 3.8^b^ |
| Lower-extremity phlebitis | 0.2 | 0.2 | 0.3 | 0.3 | 0.3^b^ | 0.5 | 0.4 | 0.4 | 0.4 | 0.4^b^ |
| Lower-extremity DVT | 2.8 | 2.8 | 3.4 | 4.7 | 3.6^b^ | 2.1 | 2.4 | 2.4 | 2.6 | 2.4^b^ |
| Superior vena cava thrombosis | 0 | 0.1 | 0.1 | 0.1 | 0.1^b^ | 0.2 | 0.1 | 0.2 | 0.2 | 0.2^b^ |
| Upper-extremity DVT | 1.3 | 1.1 | 1.1 | 1.7 | 1.3^b^ | 0.9 | 0.9 | 0.9 | 1.2 | 1.0^b^ |
| DIC | 1.4 | 1.0 | 0.9 | 1.1 | 1.1^b^ | 0.8 | 0.8 | 0.6 | 0.8 | 0.7^b^ |
| Arterial thromboembolism | 0.6 | 0.5 | 0.6 | 0.8 | 0.6^b^ | 0.7 | 1.0 | 1.0 | 0.9 | 0.9^b^ |
| Ischemic stroke | 3.1 | 3.0 | 3.4 | 4.3 | 3.5^b^ | 6.7 | 6.6 | 6.7 | 7.2 | 6.8^b^ |
| Myocardial infarction | 9.1 | 8.3 | 10.2 | 10.5 | 9.7^b^ | 10.7 | 11.7 | 1..6 | 13.2 | 12.6^b^ |
| Acute coronary syndrome | 0.1 | 0.2 | 0.2 | 0.2 | 0.2^b^ | 0.3 | 0.3 | 0.4 | 0.4 | 0.4^b^ |

^a^Admissions were limited to April 2018 or later for historical control patients and April 2020 or later for COVID-19 inpatients.

^b^*P*<0.0001 for the matched comparisons of COVID-19 ICU patients vs non-COVID-19 ICU patients.

Apr, April; COVID-19, coronavirus disease 2019; DIC, disseminated intravascular coagulation; DVT, deep vein thrombosis; ICU, intensive care unit; Mar, March; PE, pulmonary embolism; Q, quarter; Q1, January–March; Q2, April–June; Q3, July–September; Q4, October–December; VTE, venous thromboembolism.

**Supplementary Table 7** Types of anticoagulants used among hospitalized patients with COVID-19 overall and by presence or absence of a diagnosis of a thrombotic event

| **Anticoagulant Use^a^** | **All COVID-19 Inpatients** | | | **All COVID-19 ICU Inpatients** | | |
| --- | --- | --- | --- | --- | --- | --- |
|  | **Total**  **N = 546,656** | **Thrombotic Event**  **n = 56,015** | **No Thrombotic Event**  **n = 490,641** | **Total**  **n = 110,111** | **Thrombotic Event**  **n = 23,461** | **No Thrombotic Event**  **n = 86,650** |
| Any anticoagulant, % | 88.0 | 94.6 | 87.3 | 93.5 | 95.4 | 92.9 |
| Any parenteral anticoagulant^b^, % | 80.4 | 88.7 | 79.5 | 89.3 | 93.0 | 88.4 |
| Any oral anticoagulant^c^, % | 16.6 | 36.8 | 14.3 | 18.9 | 29.6 | 16.0 |
| Enoxaparin^b^, % | 64.9 | 59.1 | 65.5 | 69.0 | 62.2 | 70.8 |
| ≥ 100 mg/day | 16.4 | 33.1 | 14.5 | 31.9 | 37.0 | 30.4 |
| < 100 mg/day | 61.2 | 51.0 | 62.4 | 63.9 | 55.3 | 66.2 |
| Unknown | 0.1 | 0.2 | 0.1 | 0.1 | 0.2 | 0.1 |
| Heparin, % | 25.4 | 55.9 | 22.0 | 45.8 | 68.4 | 39.7 |
| Fondaparinux, % | 0.3 | 0.7 | 0.2 | 0.9 | 1.3 | 0.7 |
| ≥ 5 mg/day | 0.2 | 0.5 | 0.1 | 0.5 | 1.0 | 0.4 |
| < 5 mg/day | 0.2 | 0.4 | 0.2 | 0.5 | 0.7 | 0.5 |
| Unknown | 0 | 0 | 0 | 0 | 0 | 0 |
| Warfarin, % | 2.5 | 4.8 | 2.3 | 2.7 | 4.3 | 2.3 |
| Apixaban, % | 11.3 | 28.13 | 9.4 | 13.7 | 22.7 | 11.2 |
| ≤ 5 mg/day | 9.5 | 16.3 | 8.7 | 11.3 | 15.8 | 10.1 |
| > 5 and < 20 mg/day | 7.8 | 22.3 | 6.1 | 9.9 | 17.5 | 7.8 |
| ≥ 20 mg/day | 1.4 | 10.2 | 0.4 | 2.1 | 7.2 | 0.7 |
| Unknown | 0.1 | 0.2 | 0.1 | 0.1 | 0.1 | 0.1 |
| Dabigatran, % | 0.2 | 0.3 | 0.2 | 0.2 | 0.2 | 0.2 |
| ≤ 150 mg/day | 0.2 | 0.3 | 0.2 | 0.2 | 0.2 | 0.2 |
| > 150 and < 300 mg/day | 0 | 0 | 0 | 0 | 0 | 0 |
| ≥ 300 mg/day | 0.2 | 0.2 | 0.2 | 0.1 | 0.1 | 0.1 |
| Unknown | 0 | 0 | 0 | 0 | 0 | 0 |
| Edoxaban, % | 0 | 0 | 0 | 0 | 0 | 0 |
| ≤ 30 mg/day | 0 | 0 | 0 | 0 | 0 | 0 |
| > 30 and < 60 mg/day | 0 | 0 | 0 | 0 | 0 | 0 |
| ≥ 60 mg/day | 0 | 0 | 0 | 0 | 0 | 0 |
| Unknown | 0 | 0 | 0 | 0 | 0 | 0 |
| Rivaroxaban, % | 2.8 | 4.9 | 2.5 | 2.8 | 3.6 | 2.6 |
| ≤ 10 mg/day | 0.5 | 0.4 | 0.5 | 0.5 | 0.4 | 0.5 |
| > 10 and < 30 mg/day | 2.3 | 4.4 | 2.0 | 2.4 | 3.2 | 2.1 |
| ≥ 30 mg/day | 0.4 | 1.9 | 0.2 | 0.6 | 1.4 | 0.3 |
| Unknown | 0 | 0 | 0 | 0 | 0 | 0 |

^a^Patients may have received more than one anticoagulant and were counted for each anticoagulant they received for ≥ 1 day. Anticoagulants were further assessed based on the dose that is likely to be used for prophylaxis or acute treatment of thrombotic events. Because the initial dose for the acute treatment of VTE by DOACs (apixaban, dabigatran, edoxaban, rivaroxaban) tends to be higher, the analysis for DOACs was further stratified based on the dose that is likely to be used either initially or regularly for VTE treatment. In addition, patients with atrial fibrillation might have received new treatment or might have continued their anticoagulant treatment at the appropriate dose for atrial fibrillation based on the prescribing information.

^b^Parenteral anticoagulants included LMWH-enoxaparin or dalteparin, heparin, and fondaparinux. For LMWH, only enoxaparin was used in patients with COVID-19; thus, use of dalteparin is not reported.

^c^Oral anticoagulants included warfarin, apixaban, dabigatran, edoxaban, and rivaroxaban.

COVID-19, coronavirus disease 2019; DOAC, direct oral anticoagulant; ICU, intensive care unit; LMWH, low-molecular-weight heparin; VTE, venous thromboembolism.

**Supplementary Table 8** Use of anticoagulants by quarter among matched pairs of hospitalized patients with and without COVID-19^a^

|  | **Matched-pairs Comparison** | | | | | | | | | |
| --- | --- | --- | --- | --- | --- | --- | --- | --- | --- | --- |
| **Anticoagulant use**^b^ | **COVID-19 Inpatients** | | | | | **Historical Controls: Non–COVID-19 Inpatients** | | | | |
|  | **Discharged Q2 2020**  **n = 81,598** | **Discharged Q3 2020**  **n = 93,805** | **Discharged Q4 2020**  **n = 180,019** | **Discharged Q1 2021**  **n = 159,710** | **Total  Discharged  Apr 2020–Mar 2021**  **N = 515,132** | **Discharged  Q2 2018**  **n = 81,598** | **Discharged  Q3 2018**  **n = 93,805** | **Discharged  Q4 2018**  **n = 180,019** | **Discharged  Q1 2019**  **n = 159,710** | **Total  Discharged  Apr 2018–Mar 2019**  **N = 515,132** |
| Any anticoagulant, % | 85.8 | 87.2 | 88.9 | 88.0 | 87.8^e^ | 62.6 | 59.2 | 62.4 | 63.1 | 62.1^e^ |
| Any parenteral anticoagulant^c^, % | 78.4 | 81.0 | 80.2 | 79.8 | 79.9^e^ | 55.1 | 53.0 | 54.5 | 55.8 | 54.7^e^ |
| Any oral anticoagulant^d^, % | 16.0 | 13.8 | 17.3 | 17.9 | 16.6^e^ | 13.6 | 12.2 | 14.6 | 14.3 | 13.9^e^ |

^a^Admissions were limited to April 2018 or later for historical control patients and April 2020 or later for COVID-19 inpatients.

^b^Patients may have received more than one anticoagulant and were counted for each anticoagulant they received for ≥ 1 day.

^c^Parenteral anticoagulants included LMWH-enoxaparin or dalteparin, heparin, and fondaparinux.

^d^Oral anticoagulants included warfarin, apixaban, dabigatran, edoxaban, and rivaroxaban.

^e^*P*<0.0001 for the comparison of all COVID-19 inpatients vs all non-COVID-19 inpatients.

Apr, April; COVID-19, coronavirus disease 2019; Dec, December; Jan, January; Jul, July; Jun, June; LMWH, low-molecular-weight heparin; Mar, March; Oct, October; Q, quarter; Q1, January–March; Q2, April–June; Q3, July–September; Q4, October–December.

**Supplementary Table 9** Use of anticoagulants by quarter among matched pairs of hospitalized patients admitted to the ICU with and without COVID-19^a^

|  | **Matched-pairs Comparison** | | | | | | | | | |
| --- | --- | --- | --- | --- | --- | --- | --- | --- | --- | --- |
| **Anticoagulant use**^b^ | **COVID-19 ICU Inpatients** | | | | | **Historical Controls: Non–COVID-19 ICU Inpatients** | | | | |
|  | **Discharged Q2 2020**  **n = 14,193** | **Discharged Q3 2020**  **n = 17,730** | **Discharged Q4 2020**  **n = 28,971** | **Discharged Q1 2021**  **n = 25,303** | **Total  Discharged  Apr 2020–Mar 2021**  **N = 86,197** | **Discharged  Q2 2018**  **n = 14,193** | **Discharged  Q3 2018**  **n = 17,730** | **Discharged  Q4 2018**  **n = 28,971** | **Discharged  Q1 2019**  **n = 25,303** | **Total  Discharged  Apr 2018–Mar 2019**  **N = 86,197** |
| Any anticoagulant, % | 93.0 | 93.2 | 93.2 | 92.8 | 93.1^e^ | 76.7 | 75.2 | 77.1 | 78.7 | 77.1^e^ |
| Any parenteral anticoagulant^c^, % | 89.1 | 88.9 | 87.6 | 88.2 | 88.3^e^ | 72.5 | 71.8 | 73.2 | 74.8 | 73.3^e^ |
| Any oral anticoagulant^d^, % | 17.5 | 17.3 | 20.1 | 21.1 | 19.4^e^ | 15.4 | 14.6 | 16.8 | 17.2 | 16.2^e^ |

^a^Admissions were limited to April 2018 or later for historical control patients and April 2020 or later for COVID-19 inpatients.

^b^Patients may have received more than one anticoagulant and were counted for each anticoagulant they received for ≥ 1 day.

^c^Parenteral anticoagulants included LMWH-enoxaparin or dalteparin, heparin, and fondaparinux.

^d^Oral anticoagulants included warfarin, apixaban, dabigatran, edoxaban, and rivaroxaban.

^e^*P*<0.0001 for the comparison of COVID-19 ICU patients vs non-COVID-19 ICU patients.

Apr, April; COVID-19, coronavirus disease 2019; Dec, December; ICU, intensive care unit; Jan, January; Jul, July; Jun, June; LMWH, low-molecular-weight heparin; Mar, March; Oct, October; Q, quarter; Q1, January–March; Q2, April–June; Q3, July–September; Q4, October–December.
